# Heat transfer intensification of nanomaterial with involve of swirl flow device concerning entropy generation

**DOI:** 10.1038/s41598-021-91806-y

**Published:** 2021-06-14

**Authors:** Zahir Shah, M. Jafaryar, M. Sheikholeslami, Ikramullah, Poom Kumam

**Affiliations:** 1 Department of Mathematics, University of Lakki Marwat, Lakki Marwat, Khyber Pakhtun khwa 28420 Pakistan; 2 Center of Excellence in Theoretical and Computational Science (TaCS-CoE), Faculty of Science,, King Mongkut’s University of Technology Thonburi (KMUTT), 126 Pracha Uthit Rd., Bang Mod, Thung Khru, Bangkok, 10140 Thailand; 3 Renewable Energy Systems and Nanofluid Applications in Heat Transfer Laboratory, Babol Noshirvani University of Technology, Babol, Iran; 4 Department of Mechanical Engineering, Babol Noshirvani University of Technology, Babol, Islamic Republic of Iran; 5 Department of Physics, Kohat University of Science and Technology, Kohat, 26000 Khyber Pakhtunkhwa Pakistan; 6 Fixed Point Research Laboratory, Fixed Point Theory and Applications Research Group, Center of Excellence in Theoretical and Computational Science (TaCS-CoE), Faculty of Science, King Mongkut’s University of Technology Thonburi (KMUTT), 126 Pracha Uthit Rd., Bang Mod, Thung Khru, Bangkok, 10140 Thailand; 7 Department of Medical Research, China Medical University Hospital, China Medical University, Taichung, 40402 Taiwan

**Keywords:** Engineering, Mathematics and computing, Nanoscience and technology, Physics

## Abstract

The thermal features of hybrid nano-powder turbulent motion through a pipe employing helical turbulator is numerically simulated via Finite Volume Method (FVM). The hybrid nanofluid (MWCNTs + Fe_3_O_4_ + H_2_O) is obtained by uniformly dispersing MWCNTs + Fe_3_O_4_ nanomaterials in H_2_O. The characteristics features of thermal energy transfer of hybrid nanofluid are investigated by varying the pitch ratio (P) of the helical turbulator and Reynolds number (Re) of the fluid. The outputs of the study are depicted in terms of contour plots of temperature, velocity, frictional irreversibility S_gen,f_, and thermal irreversibility S_gen,th_. The variation of S_gen,f_, and S_gen,th_ with changing P and Re are also displayed by 3D plots. It is found that making the fluid more turbulent by increasing Re, the temperature of the fluid drops whereas the fluid velocity augments. The frictional irreversibility enhances, whereas the thermal irreversibility drops with the increasing turbulent motion. The decreasing P causes to drop the temperature of the higher turbulent fluid flow, while opposite effect is observed for smaller Re. The decreasing P causes to enhance the fluid mixing and thus augments the fluid velocity. S_gen,f_ and S_gen,th_ both augment with decreasing P. The comparison of current outputs with the older article shows an acceptable accuracy. The results of the present investigation will be useful in modelling of efficient thermal energy transfer systems.

## Introduction

To augment heat transfer in different process like heat exchangers, refrigeration, chemical process and automotive cooling, varieties of methods have been developed^1–3^. These methods are actually the augmenting techniques which can enhance the performance rate, reduce the system size and also minimize the working cost. The heat transfer enhancing approaches can be mainly characterized as active and passive methods. The passive technique uses turbulator such as twisted tape, spiral fins, etc. whereas active technique uses power from external source. Due to small size, simple manufacturing, high strength and low occupancy, pipes are used widely as thermal exchanger in different commercial and industrial sectors. The common widely used heat transfer ordinary single-phase fluids are oils, water, ethylene glycol, etc. The issue with using single-phase fluid is its low thermal conductivity. In the recent past, nanofluids have been developed to be used instead of ordinary single-phase fluids due to their high thermal conductivities^4–7^. Nanofluids are obtained by intermixing nanoparticles in the host fluids. The size of the nanoparticles lies in 1 to 100 nm range. Nanoparticles are made usually from metals and their oxides. Nanofluids can have tremendous thermal conductivities, which find applications for heat transfer processes in different sectors of engineering and technology^8–10^. Choi^11^ scrutinized the idea of nanomaterial as carrier fluid. Researchers have investigated different features such as shape, size, and concentration of nanomaterials for augmenting the nanofluid thermal energy transfer rate. Nowadays, hybrid nanofluids which consist of nanoparticles of more than one kind are also used for augmenting the thermal features of ordinary fluids. Khedkar et al.^12^ studied the Al2O3-H2O nanofluid thermal characteristics by using 3% volume concentration of Al2O3 nanomaterial in H2O. They obtained an increase of 16% Nu. Hilo et al.^13^ reviewed the different features of graphene nanomaterial, and described its applications in cooling phenomena. Wu et al.^14^ examined the heat transportation features of H_2_O-Al_2_O_3_ nanomaterial moving through a helical shape heat exchanger. They showed that the nanoparticles impact can be neglected on various critical Reynold numbers. They also found that nanomaterial can grow Nu up to 3.43%. Sun et al.^15^ experimentally scrutinized the performance of different nano-powders flowing through pipes having twisted tapes. They found that the system efficiency enhances with the increasing nanoparticles concentration. Chougule and Sahu^16^ performed an investigation of Al_2_O_3_ + H_2_O and CNT + H_2_O nanofluids moving through a tube containing complex tape. They found that for the twist ratio 1.5, highest heat energy transfer occurs. Mohamad et al.^17^ used FVM to examine the heat energy transfer capability of different nanomaterials types, such as ZnO, Alumina, CuO, and SiO_2_ using various volume concentrations maximum up to 4%. Their results show that SiO_2_ has the highest Nu. Labib et al.^18^ analyzed the thermal features of H_2_O-CNTs nanofluid containing Al_2_O_3_ nanoparticles. The authors reported a tremendous augment in the convection for such type of nanofluid. They found that *h* increases by 22.8% and 59.86% when 0.6 and 1.6 vol% of Al_2_O_3_ are added with 0.05 vol% of H_2_O-CNT. The thermal efficiency of radiator was examined by Prayogo et al.^19^. They investigated the cooling rate for different conditions. Garcia et al.^20^ defined the basics of helical device and used it for homogenous fluid motion in the transitional area of a pipe. They found high Nusselt number for the investigated system. Sajid and Ali^21^ presented different applications of nanomaterials in their review article. For intensification of swirl flow, Qi et al.^22^ used rotating twisted tape during nanofluid flow through a system.

The efficiency of heat transfer system deteriorates with the enhancing use of free energy available for doing useful work in the system^23–26^. Entropy analysis becomes important to investigate the thermal features of the considered system. Bejan^27^ introduced the irreversibility optimization concept during convective thermal energy transformation processes. Finding innovative kind of operating fluid was main aim of various scientists^28–30^. Ellahi et al.^31^ scrutinized the impacts of various shapes nanomaterial using irreversibility optimization. Miroshnichenko et al.^32^ analyzed the nanofluid convective magnetized motion from a trapezoidal cavity. Their study displays that the value of Nu drops with the increasing MHD, while grows with the enhancing concentration of nano-powders. Shafee et al.^33^ performed numerical simulation study of nanofluid turbulent motion through entropy analysis. During their investigations, they depicted that the thermal energy transfer improves with the augmenting turbulator height and exergy loss is related inversely with input power. Feroz et al.^34^ employed the effective conductivity model to investigate the CNTs nanofluid motion in a rotating channel by considering Ion slip and Hall effects. They applied entropy optimization principle to model the thermal characteristics of the nanofluid motion. Elahi et al.^35^ examined the nanofluid peristaltic motion through permeable enclosure using entropy optimization principle.

Motivated from the above studies, it is planned to investigate the thermal energy transfer features of hybrid nanofluid turbulent motion using entropy optimization. The innovation of the current article is the second law investigation of the hybrid nanomaterial migration by inserting a helical turbulator in tube. The helical turbulator is used to make the flow more swirling and generating secondary flow. Among different geometric parameters of the helical turbulator, the impact of varying pitch ratio (P) over the heat transfer characteristics is investigated in the current work. The model relations are numerically solved through FVM. The outputs of the study are explained through contour and 3D plots. This research work has potential applications in heat exchangers, refrigeration, automotive cooling, and different physical and chemical processes. This article is organized in the following way. The physical model of the study is explained in “Physical model” section. The hybrid nanofluid motion is mathematically simulated in part 3. The numerical outputs were explained in view of contour and 3D plots in part 4. At the end the highlights of the current investigations are displayed.

## Physical model

**Figure 1:**
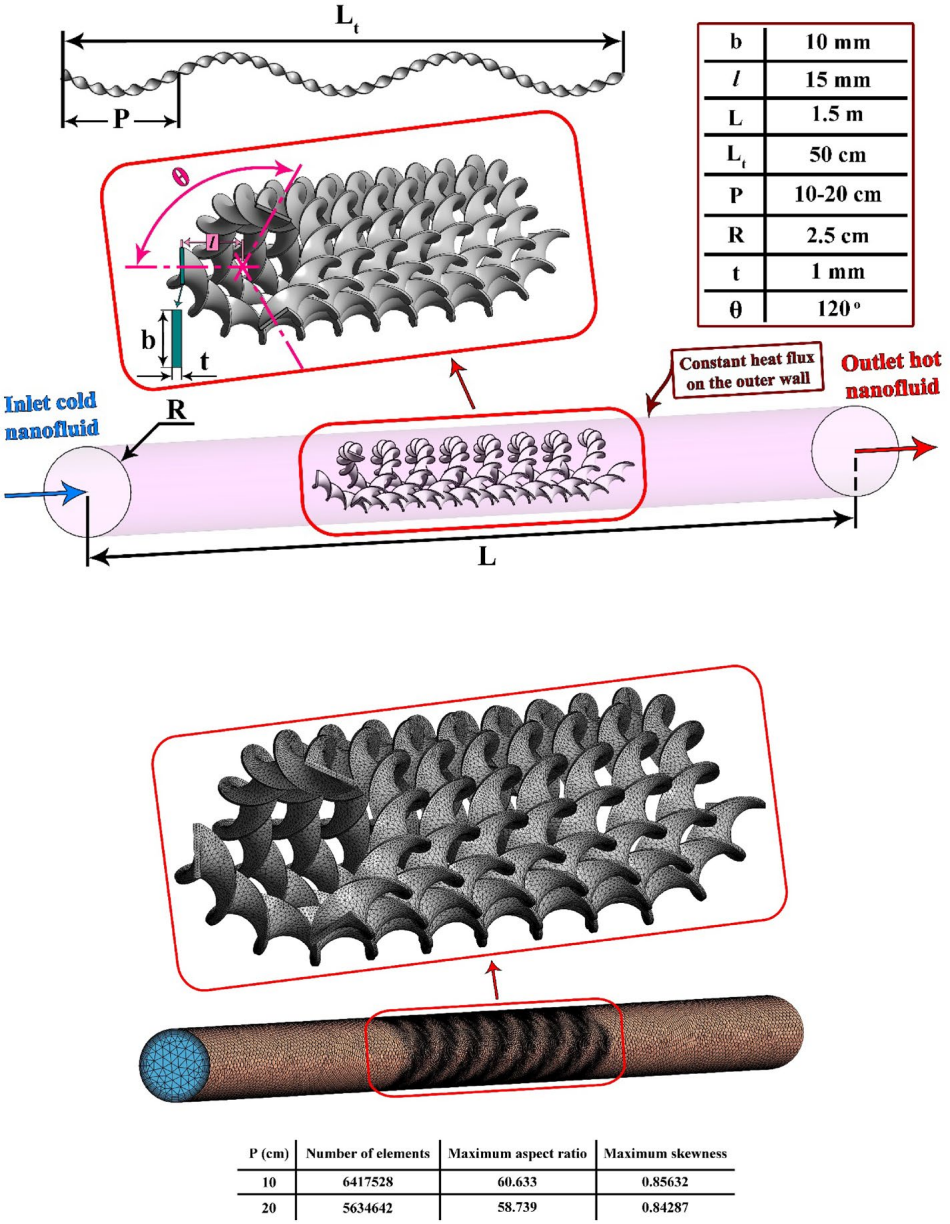
Pipe with complex tapes and relevant grid.

Figure 1 exhibits the schematics of the present work. It comprises of a tube filled with (MWCNTs + Fe_3_O_4_ + H_2_O) hybrid nanofluid. A helical turbulator is used to make the nanofluid motion turbulent and more swirling inside the tube. The sample mesh is also shown in Fig. 1. The boundary restrictions satisfied by the fluid motion are described in this figure. Table 1 displays the physical features of the understudied nanofluid^36^.

**Table 1:** Feature of operating fluid^36^.

$\phi$	$\mu$ (mPa s)	$C_{p}$_(j/kgk)_	$k$_(W/m k)_	$\rho$_(kg/m_^3^_)_
0.003	1.01	4183.99	0.6856	1010.04
0	0.79	4182	0.602	998.5

## Problem formulation

Forced convection turbulent hybrid nanofluid motion through a heat exchanger can be mathematically formulated as^37,38^

1 $$\frac{{\partial \left( {u_{i} } \right)}}{{\partial x_{i} }} = 0$$

2 $$\frac{\partial }{{\partial x_{j} }}\left( {u_{j} u_{i} \rho_{nf} } \right) = - \frac{\partial p}{{\partial x_{i} }} + \frac{\partial }{{\partial x_{j} }}\left( {\left( {\frac{{\partial u_{i} }}{{\partial x_{j} }} + \frac{{\partial u_{j} }}{{\partial x_{i} }}} \right)\mu_{nf} } \right) + \frac{\partial }{{\partial x_{j} }}\left( { - \rho_{nf} \overline{{u_{j}^{\prime } u_{i}^{\prime } }} } \right)$$

3 $$\frac{\partial }{{\partial x_{i} }}\left( {\rho_{nf} Tu_{i} } \right) = \frac{\partial }{{\partial x_{i} }}\left( {\left( {\Gamma + \Gamma_{t} } \right)\frac{\partial T}{{\partial x_{i} }}} \right),\,\,\,\,\Gamma \left( { = \mu_{nf} /\Pr_{nf} } \right),\,\,\,\,\Gamma_{t} \left( { = \mu_{t} /\Pr_{t} } \right)$$

$\rho_{nf} \overline{{u^{\prime}_{j} u^{\prime}_{i} \,}}$ and $\mu_{t}$ are given as^38^:

4 $$- \rho_{nf} \overline{{u^{\prime}_{i} \,u^{\prime}_{j} }} = \left( {\frac{{\partial u_{i} }}{{\partial x_{j} }} + \frac{{\partial u_{j} }}{{\partial x_{i} }}} \right)\mu_{t} - \frac{2}{3}\rho_{nf} k\delta_{ij} - \frac{2}{3}\mu_{t} \frac{{\partial u_{k} }}{{\partial x_{k} }}\delta_{ij}$$

5 $$\mu_{t} = \frac{1}{\varepsilon }k^{2} C_{\mu } \rho_{nf}$$

Turbulent model has been added two scalars^37^:

6 $$\frac{\partial }{{\partial x_{j} }}\left( {\left( {\frac{{\mu_{t} }}{{\sigma_{k} }} + \mu_{nf} } \right)\frac{\partial k}{{\partial x_{j} }}} \right) - \rho_{nf} \varepsilon + G_{k} = \frac{\partial }{{\partial x_{i} }}\left( {u_{i} \rho_{nf} k} \right),\,\,\,\,G_{k} = - \rho_{nf} \,\overline{{u^{\prime}_{j} u^{\prime}_{i} \,}} \frac{{\partial u_{j} }}{{\partial x_{i} }}$$

7 $$\frac{\partial }{{\partial x_{i} }}\left( {u_{i} \rho_{nf} \varepsilon } \right) = \frac{\partial }{{\partial x_{j} }}\left( {\frac{\partial \varepsilon }{{\partial x_{j} }}\left( {\frac{{\mu_{t} }}{{\sigma_{\varepsilon } }} + \mu_{nf} } \right)} \right) + \frac{\varepsilon }{k}G_{k} C_{1\varepsilon } - \rho_{nf} \frac{{\varepsilon^{2} }}{k}C_{2\varepsilon }$$

Here,

8 $$\,C_{1\varepsilon } = 1.42,\,\,C_{\mu } = 0.0845,\,\,C_{2\varepsilon } = 1.68, \, \Pr_{t} = 0.85, \, \sigma_{k} = 1, \, \sigma_{\varepsilon } = 1.3.\,$$

This study uses the empirical data of the mixture of (MWCNT + Fe_3_O_4_ + H_2_O) properties as illustrated in Table 1^36^. The nanofluid properties are described by the relations^36^:

9 $$\rho_{nf} \beta_{nf} = \left( {1 - \varphi } \right)\left( {\rho \beta } \right)_{bf} + \varphi \left( {\rho \beta } \right)_{np} {, }\beta_{np} = \frac{{\beta_{MWCNT} \varphi_{MWCNT} + \beta_{{Fe_{3} O_{4} }} \varphi_{{Fe_{3} O_{4} }} }}{{\varphi_{MWCNT} + \varphi_{{Fe_{3} O_{4} }} }}$$

Further details can be found in the previous publication^33^.

Here, z = 0 and z = L satisfy the conditions:

10 $$v_{i} = 0, \, w_{i} = cte, \, u_{i} = 0, \, I = 0.16(Re)^{{\frac{ - 1}{8}}} , \, T_{i} = cte$$

11 $$\frac{{\partial {\text{u}}}}{{\partial {\text{z}}}} = \frac{{\partial {\text{v}}}}{{\partial {\text{z}}}} = \frac{{\partial {\text{T}}}}{{\partial {\text{z}}}} = \frac{{\partial {\text{w}}}}{\partial z} = 0$$

The definitions of the irreversibly terms $S_{gen,total} ,S_{gen,th} ,S_{gen,f}$ are^33^:

12 $$\begin{gathered} S_{gen,total} = S_{gen,th} + S_{gen,f} \hfill \\ = \frac{{k_{nf} }}{{T^{2} }}\left[ {\left( {\frac{\partial T}{{\partial x}}} \right)^{2} + \left( {\frac{\partial T}{{\partial y}}} \right)^{2} + \left( {\frac{\partial T}{{\partial z}}} \right)^{2} } \right] + \frac{{\mu_{nf} }}{T}\left\{ \begin{gathered} 2\left[ {\left( {\frac{{\partial u_{x} }}{\partial x}} \right)^{2} + \left( {\frac{{\partial u_{y} }}{\partial y}} \right)^{2} + \left( {\frac{{\partial u_{z} }}{\partial z}} \right)^{2} } \right] + \hfill \\ \left( {\frac{{\partial u_{x} }}{\partial y} + \frac{{\partial u_{y} }}{\partial x}} \right)^{2} + \left( {\frac{{\partial u_{x} }}{\partial z} + \frac{{\partial u_{z} }}{\partial x}} \right)^{2} + \left( {\frac{{\partial u_{y} }}{\partial z} + \frac{{\partial u_{z} }}{\partial y}} \right)^{2} \hfill \\ \end{gathered} \right\} \hfill \\ \end{gathered}$$

**Figure 2:**
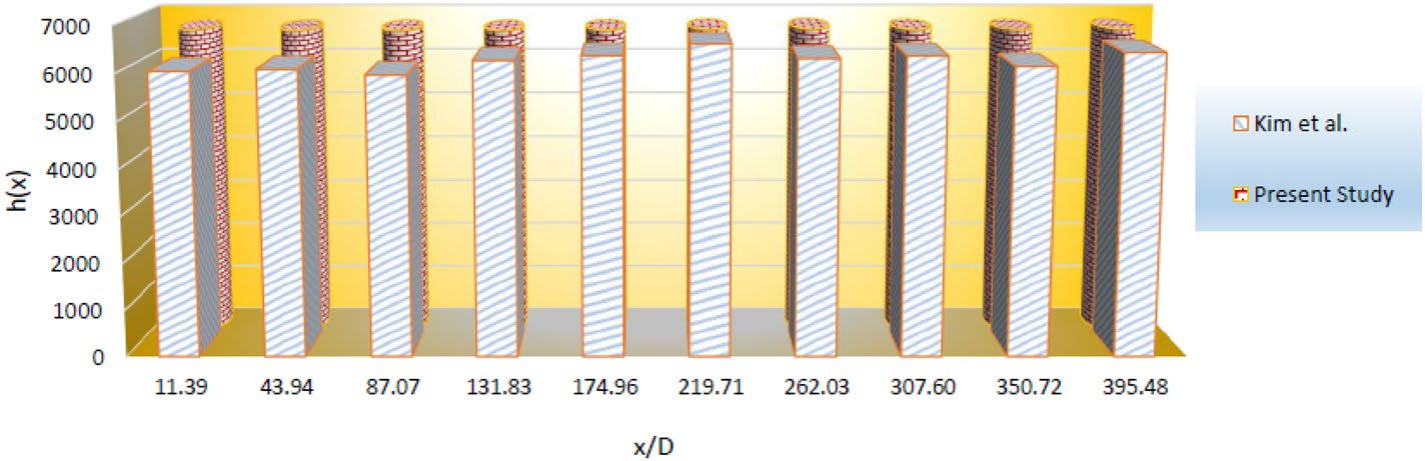
Showing acceptable accuracy of code^39^.

The setting of ANSYS Fluent is the same of Ref.^29^. Figure 2 demonstrates the validation of current study by comparing it with the work in^35^. The comparison shows an acceptable accuracy.

## Results and discussion

In current research the hybrid nano-powder migration through a pipe having a helical turbulator is simulated numerically via FVM. The helical turbulator causes to make the fluid motion more turbulent and also causes secondary fluid flow. The tube is under effect of constant heat flux. The entropy analysis is applied to examine the hydrothermal features of the fluid motion. The hybrid nanofluid (MWCNT + Fe_3_O_4_ + H_2_O) is chosen as working substance considering single phase model to estimate its properties. The different aspects of the turbulent fluid motion are studied by depicting contour graphs of temperature, frictional and thermal irreversibilities (S_gen,f_, S_gen,th_) as function of varying pitch ratio (P) of the helical turbulator and enhancing Reynolds number (Re) of the hybrid nanofluid motion. The 3D plots are plotted which display the functional dependence of S_gen,f_ and S_gen,th_ with augmenting pitch ratio (P) and enhancing Re associated with higher values of the input power.

**Figure 3:**
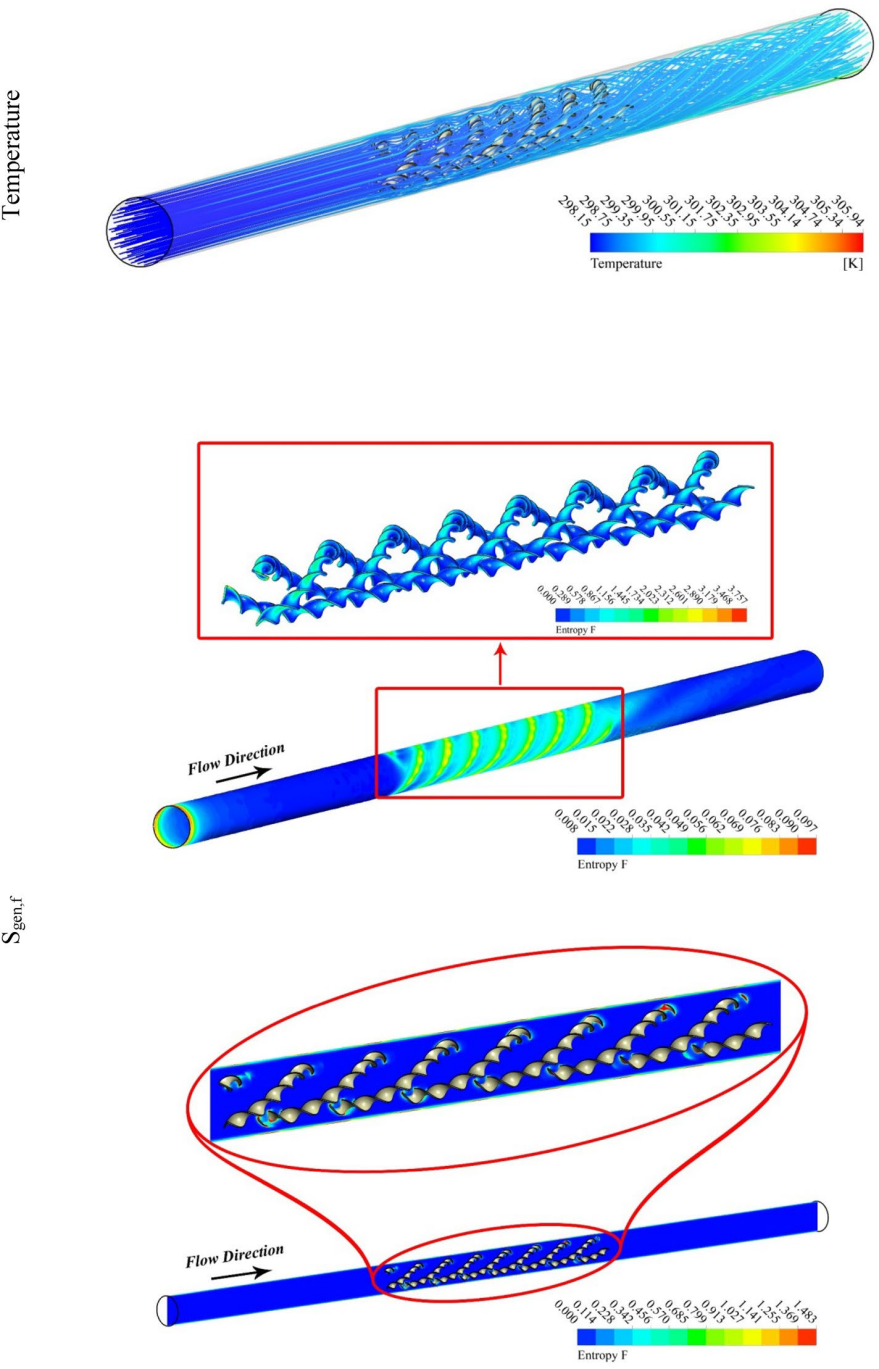

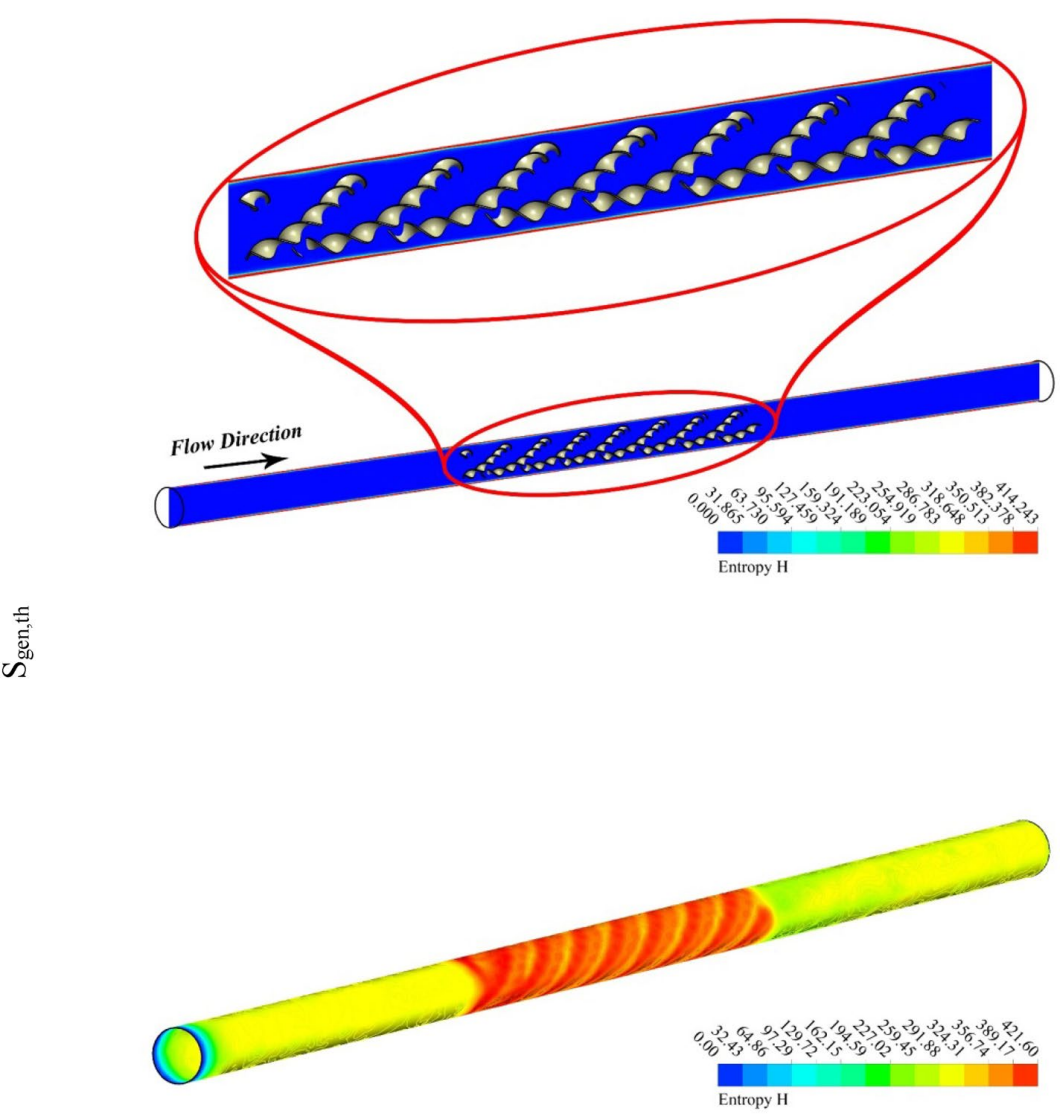
Temperature, entropy generation distributions at P = 0.2 m, Re = 5000.

**Figure 4:**
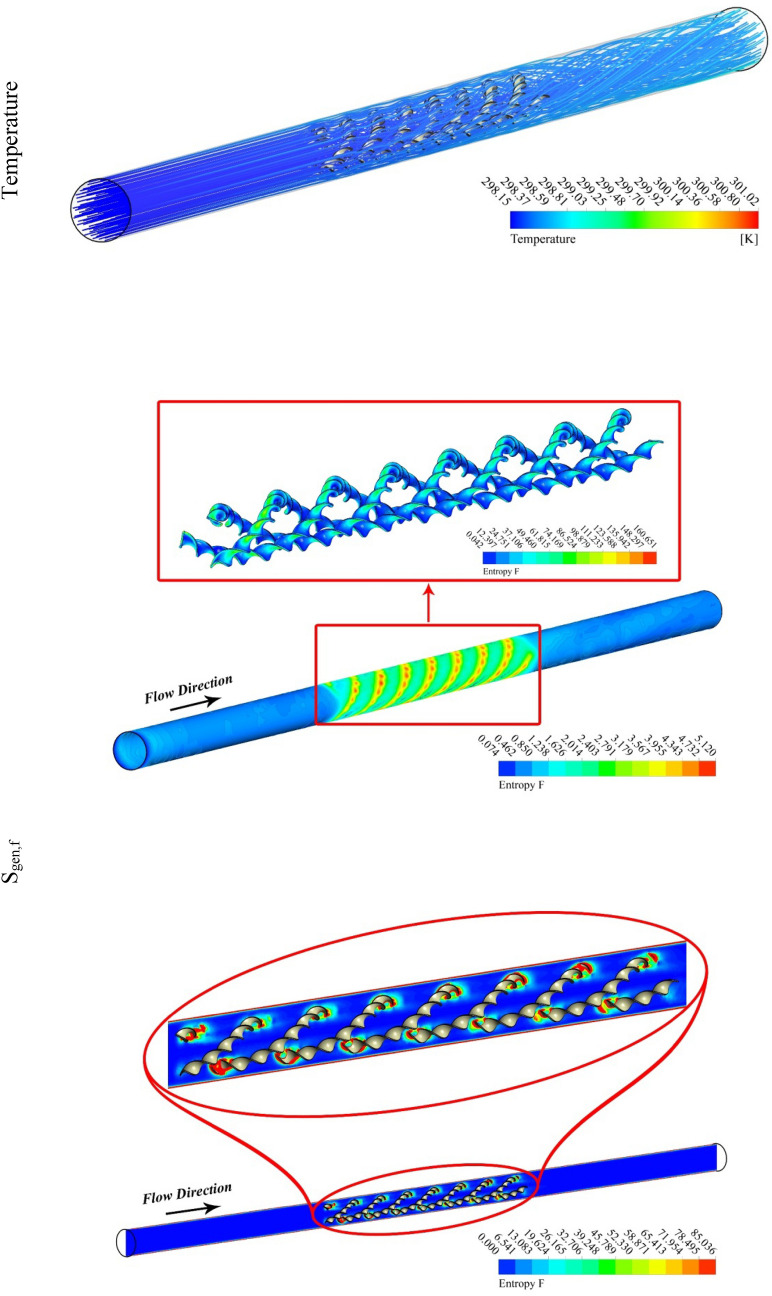

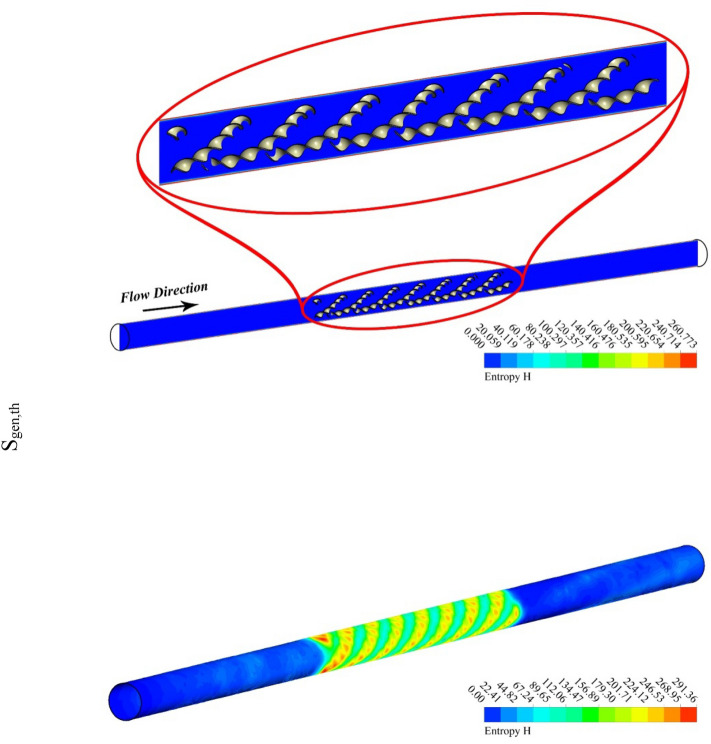
Temperature, S_gen_ distributions at Re = 20,000, P = 0.2 m.

**Figure 5:**
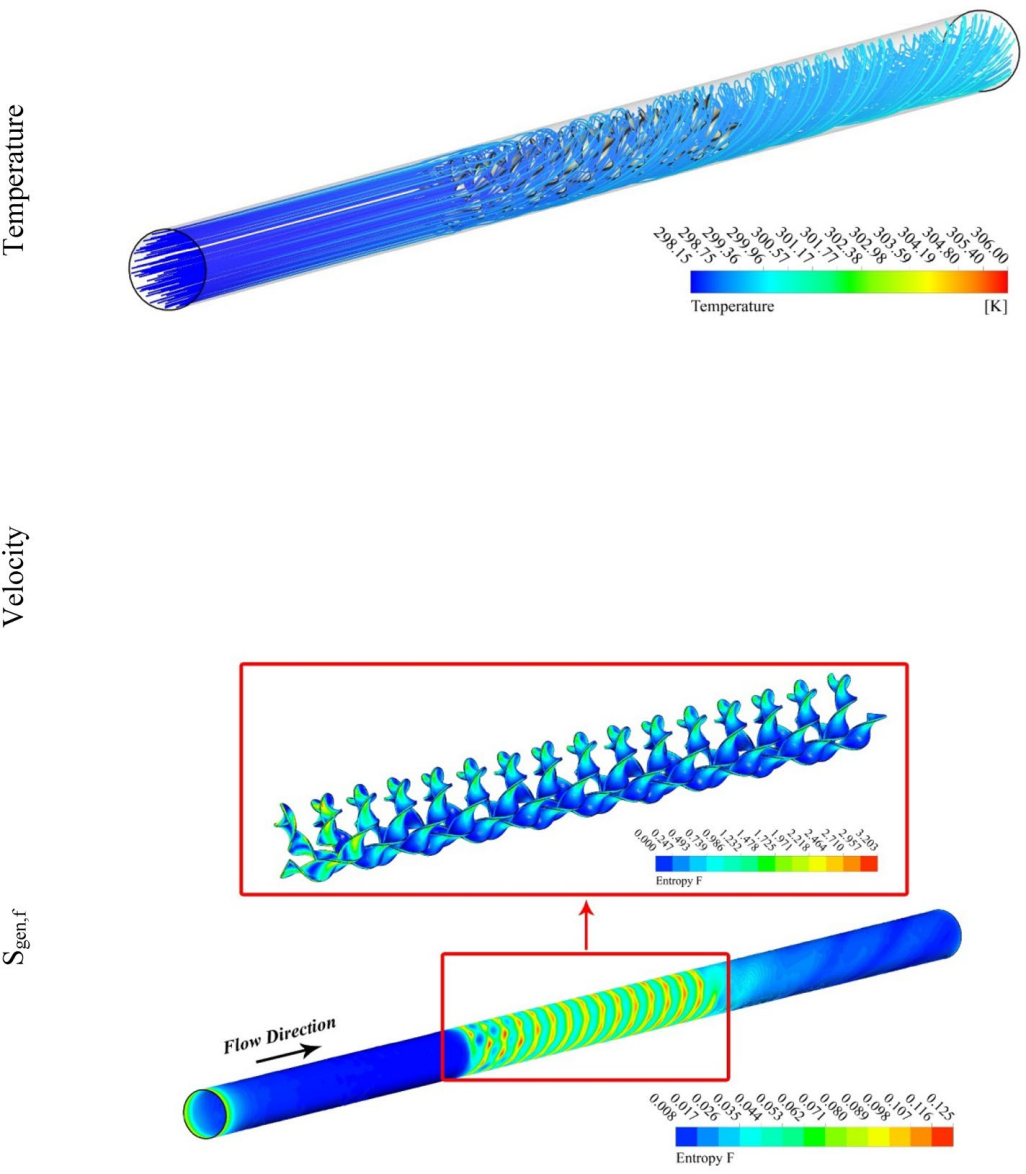

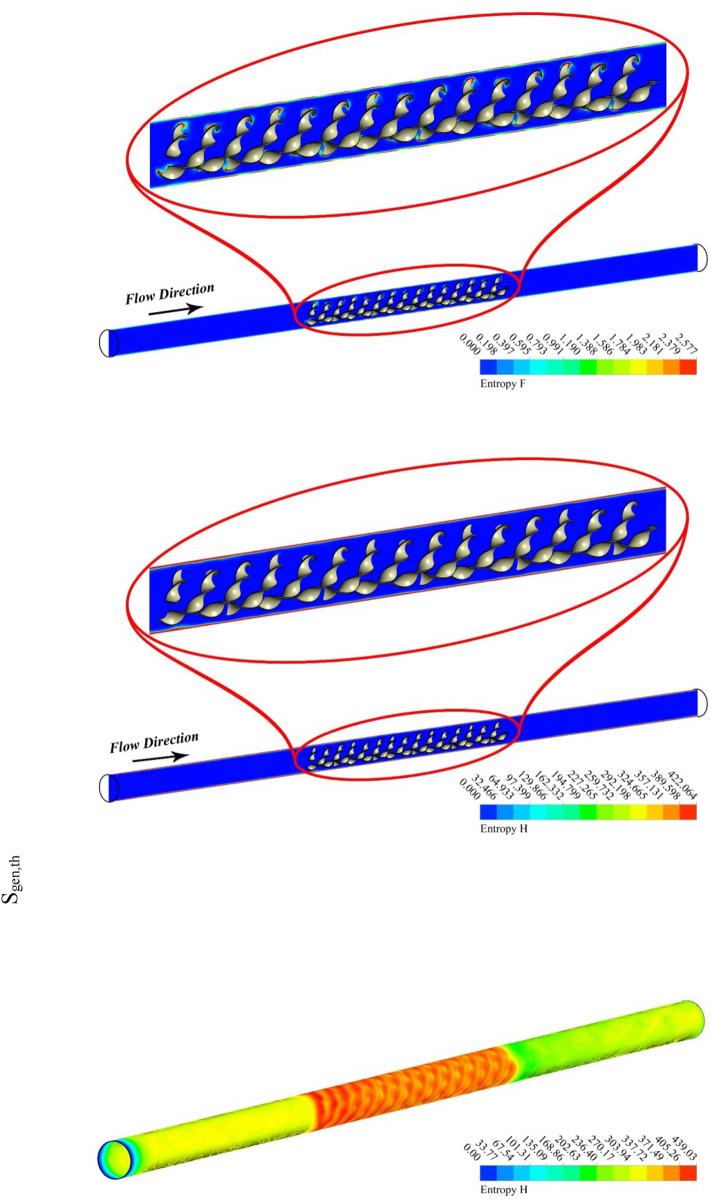
Temperature, entropy generation distributions at Re = 5000, *P* = 0.1 m.

**Figure 6:**
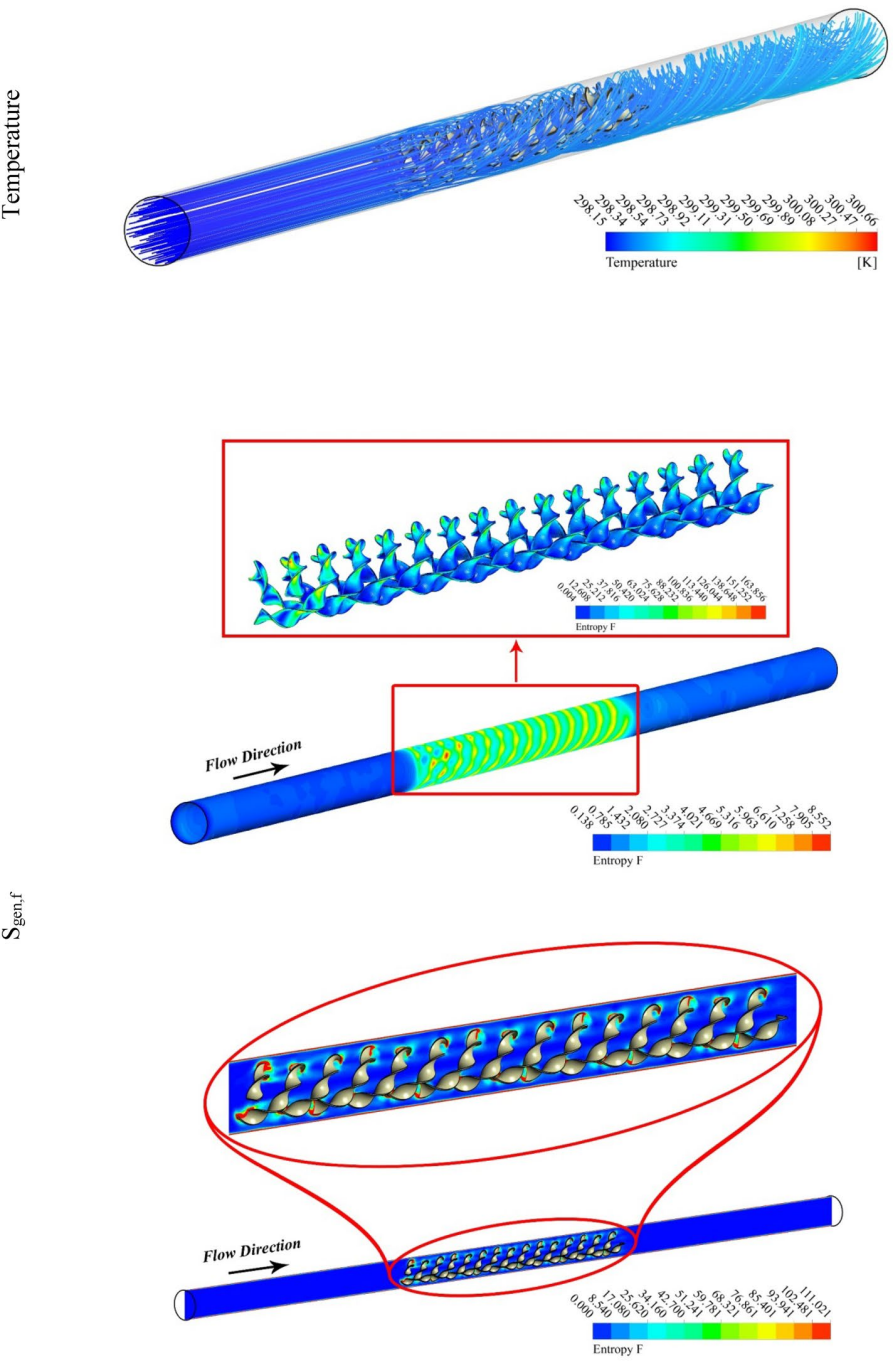

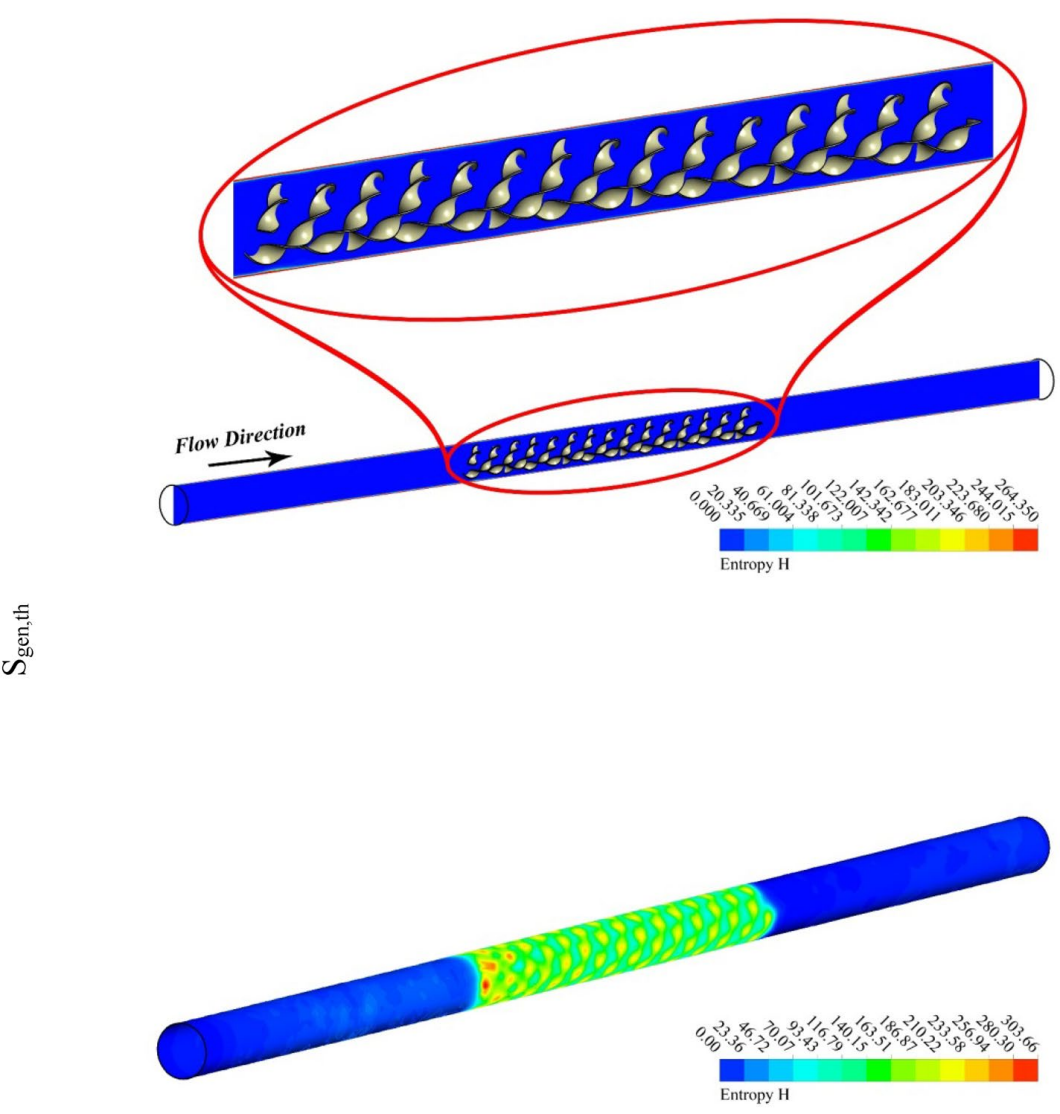
S_gen_, and temperature contours at Re = 20,000, *P* = 0.1 m.

The outputs of the current study were explained by depicting the contour and 3D plots showing the variation of hybrid nanofluid characteristics with changing pitch ratio of the helical turbulator and enhancing turbulent motion associated with higher values of Reynolds number. Figures 3, 4, 5 and 6 show the contour plots of temperature, frictional irreversibility (S_gen,f_), and thermal irreversibility ( S_gen,th_) at varying values of Reynold number (Re) and pitch ratio (P). In Figs. 3 and 4, the values of Re are taken as 5000 and 20,000, whereas the value of P is taken as 0.2. Similarly, in Figs. 5 and 6, the values of Re are taken as 5000, and 20,000, whereas the value of P is used as 0.1. The contour plots of temperature show that the fluid temperature drops with the enhancement in Re. Thus the enhancing turbulent motion drops the temperature of the hybrid nanomaterial. The velocity of the fluid enhances with the augmenting Re and this increase in the fluid velocity with rising Re is attributed to the augmenting input power. The contour plots for frictional irreversibility shows that, S_gen,f_ enhances due to enhanced swirling flow as a result of the insertion of helical turbulator. The increase in Reynolds number due to the enhancing input power causes to augment the entropy creation tremendously due to higher frictional motion. The enhancing swirling flow also results a decrease in S_gen,th_ owing to decrement of ∇T between the fluid and wall of the tube. The enhancement in Reynolds number due to increase in the input power also drops the entropy generation as an output of decrease in the ∇T that exists among the fluid and tube wall.

Figures 5 and 6 are plotted for smaller pitch ratio (*P* = 0.1) as compared to the previous value for P in Figs. 3 and 4. The values of Re used in these figures are 5000 and 20,000, respectively. It is observed that the enhancing Re results reduction in the fluid temperature. It is also cleared that the decrease in *P* also drops the temperature of the fluid. Thus the augmenting swirling flow due to smaller pitch ratio and the generation of secondary flow result in the reduction of the fluid temperature. The contour plots for the velocity shows an enhancement in the velocity with the increasing Re. This is obvious that fluid velocity shall be larger at higher input power (larger Re). Furthermore, the turbulator with smaller P also enhances the fluid velocity due to high swirling motion. The distributions of S_gen,f_ display that the entropy development linked with resistance of fluid because nanomaterial motion augments with the increasing Re as a result of enhancing friction associated with the higher fluid velocities. Furthermore, S_gen,f_ augments with the decreasing P except at Re = 5000. The entropy generation related with ∇T exists among the fluid and walls of the container decreases with the augmenting input power (higher Re). This displays that the temperature gradient exists between fluid and wall of the tube drops with the enhancing turbulent motion.

The following formulae are used for computing $S_{gen,f} ,S_{gen,th}$:

13 $$S_{gen,f} = 0.038 - 4.8 \times 10^{ - 3} \,P + 0.035{\text{Re}}^{*} - 4.47 \times 10^{ - 3} \,P\,{\text{Re}}^{*}$$

14 $$S_{gen,th} = 6.98 + 0.31\,P - 5.9{\text{Re}}^{*} - 0.23\,P\,{\text{Re}}^{*}$$

It is evident from Eqs. (13) and (14) that the entropy generation due to hybrid nanofluid frictional motion ($S_{gen,f}$) and due to the existence of ∇T depends upon the pitch ratio (P) of the helical turbulator and modified Reynolds number ${\text{Re}}^{*}$. The functional dependence of the entropy generation with varying P and ${\text{Re}}^{*}$ is explained by plotting the 3D plots between these quantities.

**Figure 7:**
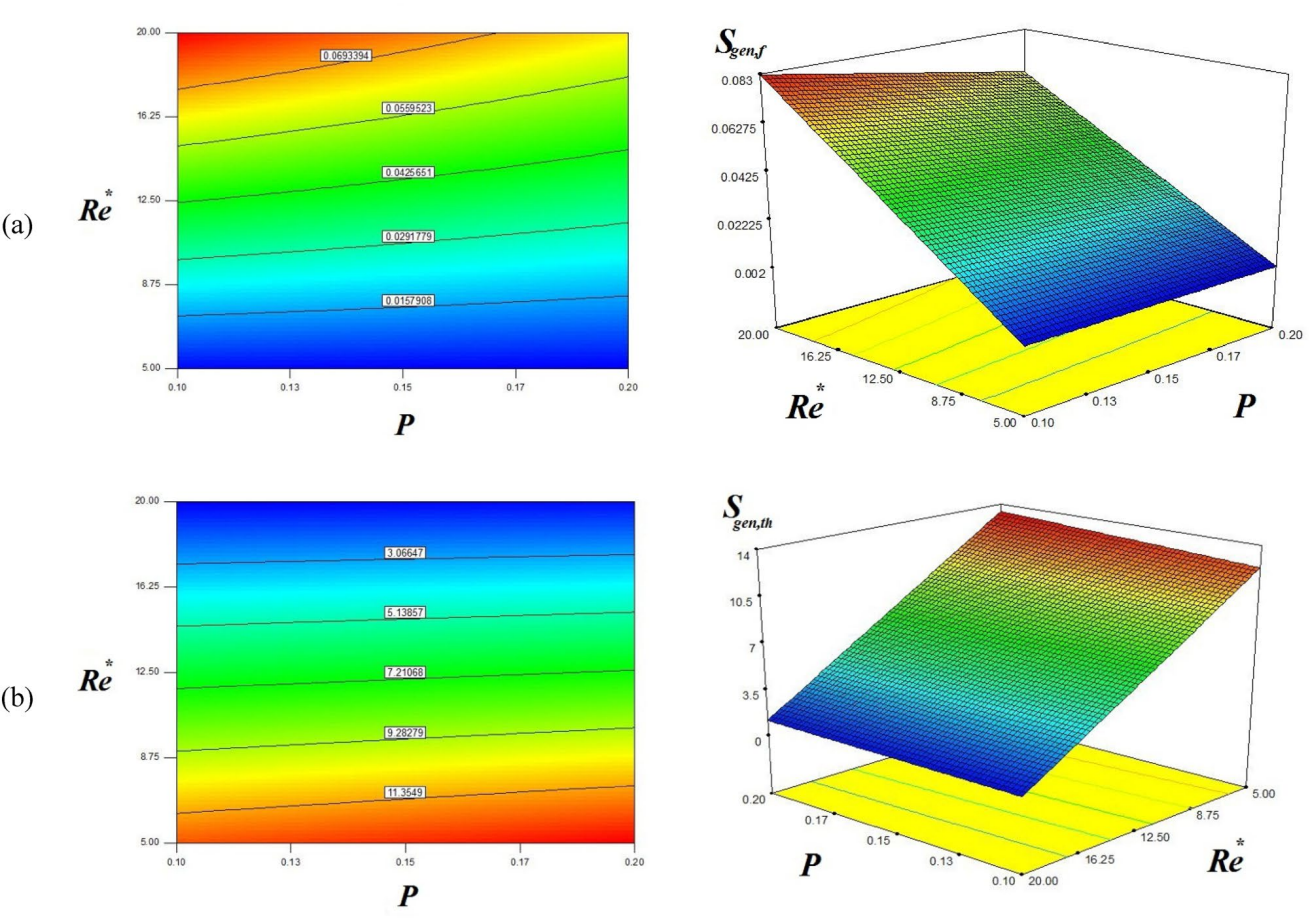
Friction and thermal entropy generations for various *Re* and *P*.

Figure 7 depicts the variation of S_gen,f_ and S_gen,th_ with varying values of Reynolds number (Re^*^) and pitch ratio (P) of the helical turbulator. The figure displays that S_gen,f_ augments with enhancing Re^*^, while drops with increasing P. We see that the rate of enhancement of S_gen,f_ with rising Re^*^ is much larger than its rate of drop with increasing P. This shows that the higher the hybrid nanofluid turbulent motion, the larger S_gen,f_ will be achieved. The 3D plots for S_gen,th_ display that the entropy generation variation owing to the temperature difference. It drops with the increasing input power (augmenting Re^*^). We also observed that S_gen,th_ drops with the enhancing P. But this rate of drop of S_gen,th_ with rising P is much smaller than the drop with rising Re^*^.

## Conclusion

The hybrid nanofluid turbulent motion through a pipe in the presence of constant heat flux is numerically simulated in this study. A helical turbulator is employed to make the fluid motion a swirling one and to generate secondary flow. The modeled formulation consisting of flow and temperature equations are solved using FVM. The forced convective features of the flow are analyzed through entropy optimization. The impacts of varying pitch ratio of the helical turbulator (P) and Reynolds number (Re) of the fluid are investigated over the forced convective characteristics of the hybrid nanofluid motion through the heat exchanger. The results are displayed through contour and 3D plots. The comparison with the published work ascertains an acceptable accuracy. It is perceived that the fluid temperature drops while velocity enhances with increasing Re. The thermal irreversibility S_gen,th_ associated with the temperature gradient among the working fluid and wall drops, while the frictional irreversibility S_gen,f_ enhances with the increasing turbulence (higher Re) of the hybrid nanofluid. The decreasing pitch ratio (P) of the helical turbulator enhances the fluid velocity for the entire turbulent flow and drops the fluid temperature at higher Re. The entropy generation augments with the decreasing values of P.

## Data Availability

The data that support the findings of this study are available from the corresponding author upon reasonable request.
